# Sound-contingent visual motion aftereffect

**DOI:** 10.1186/1471-2202-12-44

**Published:** 2011-05-15

**Authors:** Souta Hidaka, Wataru Teramoto, Maori Kobayashi, Yoichi Sugita

**Affiliations:** 1 Department of Psychology, Rikkyo University, 1-2-26, Kitano, Niiza-shi, Saitama, 352-8558 Japan; 2 Department of Psychology, Graduate School of Arts and Letters, Tohoku University, 27-1, Kawauchi, Aoba-ku, Sendai, Miyagi, 980-8576 Japan; 3 Research Institute of Electrical Communication, Tohoku University, 2-1-1, Katahira, Aoba-ku, Sendai, Miyagi, 980-8577 Japan; 4 Neuroscience Research Institute, National Institute of Advanced Industrial Science and Technology (AIST), 1497-1 Tsukuba, Ibaraki, 300-4201 Japan

## Abstract

**Background:**

After a prolonged exposure to a paired presentation of different types of signals (e.g., color and motion), one of the signals (color) becomes a driver for the other signal (motion). This phenomenon, which is known as contingent motion aftereffect, indicates that the brain can establish new neural representations even in the adult's brain. However, contingent motion aftereffect has been reported only in visual or auditory domain. Here, we demonstrate that a visual motion aftereffect can be contingent on a specific sound.

**Results:**

Dynamic random dots moving in an alternating right or left direction were presented to the participants. Each direction of motion was accompanied by an auditory tone of a unique and specific frequency. After a 3-minutes exposure, the tones began to exert marked influence on the visual motion perception, and the percentage of dots required to trigger motion perception systematically changed depending on the tones. Furthermore, this effect lasted for at least 2 days.

**Conclusions:**

These results indicate that a new neural representation can be rapidly established between auditory and visual modalities.

## Background

New neural representations can be established even in the adult brain: After an exposure to repeated alternations of red contracting and green expanding spirals, the red stationary spiral appeared to be expanding, while the green stationary spiral appeared to be contracting. This phenomenon is called as contingent motion aftereffect and has been reported only in visual [1,2] and auditory [3] domains. However, perceptual events can also involve multiple sensory modalities simultaneously; for instance, visual movements often accompany a corresponding sound in the real world. Thus, the perceptual systems adequately integrate diverse information from different sensory modalities in order to create a robust perception [4]. Therefore, it is possible that contingent motion aftereffects also occur across sensory modalities. Here, we demonstrate visual motion aftereffects contingent on arbitrary sounds.

**Figure 1:**
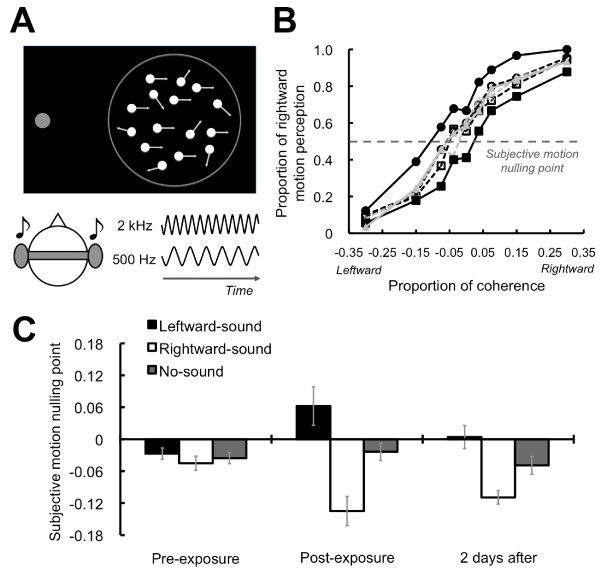
**Schematics of stimuli and results for exposure effects**. (A) Visual global motion display was presented with a fixation circle. Tones of 2 kHz or 500 Hz were delivered through headphones. (B) Psychometric functions. In horizontal axis, negative values indicate leftward visual motion, and positive values indicate rightward motion. Each symbol represents the conditions. Circle: rightward-sound, square: leftward-sound, and triangle: no-sound condition. Filled symbols and solid lines indicate after-exposure data, while open symbols and dashed lines indicate pre-exposure data. Point of 50% responses was estimated as subjective motion nulling points (SMNPs). (C) SMNPs. Error bars denote the standard error of the mean.

We compared the results of test sessions conducted before and after an exposure session. In our test session, a visual global motion display was presented to the participants. In this display, local motion signals formed of dynamic dot patterns were combined to produce a coherent global motion perception. In each trial, we manipulated the dots' coherence: 0%, 3.75%, 7.5%, 15%, or 30% of dots moved either leftward or rightward, and the other dots moved in random directions (Figure 1A). The onset of the display was synchronized with a pure tone of either high (2 kHz) or low (500 Hz) frequency. In our 3-minutes exposure session, motion display with 100% coherence was presented. The leftward motion was synchronized with the high-frequency tone (leftward-sound condition), and the rightward motion was synchronized with the low frequency tone (rightward-sound condition) or vice versa. During display, each visual motion direction was alternated. We found that the tones systematically changed the visual motion perception after the exposure.

## Methods

### Participants and apparatus

Nine participants, including the authors, had normal or corrected-to-normal vision and normal hearing. Apart from the authors, the participants were naïve to the purpose of the experiments. Informed consent was obtained from each participant before conducting the experiments. All procedures were approved by the local ethics committee of Tohoku University.

Visual stimuli were presented on a 24-inch CRT display (refresh rate: 60 Hz) with a viewing distance of 1 m. Auditory stimuli were generated digitally (sampling frequency: 44.1 kHz) and delivered via headphones. The synchronization of the visual and auditory stimuli was confirmed using a digital oscilloscope. The participants were instructed to place their heads on a chin rest, and the experiments were conducted in a dark room.

### Stimuli

for visual fixation, a red circle (diameter: 0.4 deg; luminance: 17.47 cd/m^2^) was presented on a black background. A global motion display containing 300 white dots (5.12 cd/m^2^) was presented as visual stimuli, on the right of the fixation circle. Each dot was 0.25 deg in diameter and was randomly located within 5 deg in diameter of an invisible circular window. The global motion display was presented at an eccentricity of 5 deg. The target motion signal was presented for 500 ms, and the dots coherence was manipulated: 3.75%, 7.5%, 15%, or 30% of dots moved either leftward or rightward as the target direction, while the remaining dots moved in random directions except for the target motion direction; 0% coherence of the moving dots was also included. The lifetime and velocity of each dot was 12 frames and 2.0 deg/s, respectively. Auditory stimulus (85 dB SPL, 500 ms in duration, and 5 ms rise and fall time) was either a high (2 kHz) or low (500 Hz) frequency tone.

### Procedure

The experiment consisted of 3 sessions--pre-test, exposure, and post-test. In the exposure session, global motion display with 100% coherence was presented. The duration of the display was 500 ms. For 5 participants, the onset of the leftward motion was synchronized to a tone burst (500 ms in duration) of high (2 kHz) frequency (leftward-sound condition), while the rightward motion was synchronized with that of the low (500 Hz) frequency (rightward-sound condition). The opposite pairing was used for the remaining 4 participants. The participants were instructed to look intently at the fixation. The presentation of the paired visual and auditory stimuli was repeated 360 times so that it lasted for 3 minutes. The visual motion directions were alternated during the presentation.

In the pre- and post-test sessions, discriminate thresholds for motion direction were measured using the method of constant stimuli. In each trial, the coherence of global motion display was randomly assigned. The onset of the display was synchronized with the tone burst of the high or low frequency. The no-sound condition was also tested. The participants were asked to judge whether the visual stimulus moved leftward or rightward. Each pre- and post-test session consisted of 270 trials; 9 coherences of moving dots × 3 auditory conditions (2 sound frequencies and 1 no-sound condition) × 10 repetitions. Each condition was randomly presented and counterbalanced among the trials. It took almost 10 minutes to complete each test session.

## Results

### Effects of the exposure

We plotted the proportion of rightward motion perception against the dots' coherences as psychometric functions. Before the prolonged exposure to the tones and visual motion, the psychometric functions in each condition were almost identical (Figure 1B). To determine subjective motion nulling points (SMNPs), we estimated the 50% point of rightward motion perception by fitting a cumulative normal-distribution function to each participant's psychometric function (Figure 1C). We confirmed that the tones did not affect visual motion perception at all. However, after the exposure, they remarkably affected visual motion perception. In the post-exposure test session, the psychometric function shifted to rightward visual motion in the leftward-sound condition and to leftward visual motion in the rightward-sound condition (Figure 1B, C). These data patterns showed that the tones paired with the rightward/leftward visual motion perceptually suppressed the opposite global visual motion and enhanced the consistent motion perception of the paired motion information. A two-way repeated measures analysis of variance (ANOVA) with tests (2; pre/post) × auditory conditions (3) showed the significant interaction effect between the factors (*F*(2 16) = 10.25, *p *< .005). Regarding a simple main effect of the auditory conditions in the post-test (*F*(2, 32) = 24.18, *p *< .001), a post hoc test (Tukey's HSD) revealed that the SMNPs were different among the auditory conditions (*p *< .05). In contrast, the simple main effect of the auditory conditions in the pre-test was not significant (*F*(2, 32) = .20, *p *= .82). These results indicate a robust sound-contingent visual motion aftereffect; sounds can induce visual motion perception for the global motion stimuli in the same direction as the exposed stimuli.

### Long-lasting effect of the exposure

It is well known that contingent aftereffects persist for a long time [1,3,5]. To estimate the persistence of the audiovisual associations in the sound-contingent visual motion aftereffect, we conducted the post-test session 2 days after the exposure (Figure 1C). Again, the ANOVA showed the significant interaction effect between the factors (*F*(2 16) = 8.18, *p *< .005). Concerning a simple main effect of the auditory conditions in the post-test (*F*(2, 32) = 21.55, *p *< .001), the post hoc test revealed that the SMNPs were different among the auditory conditions (*p *< .05). The simple main effect of the auditory conditions in the pre-test did not reach significance (*F*(2, 32) = .53, *p *= .59). These results indicate that the effect of the exposure lasted for at least 2 days.

### Selectivity of visual field

**Figure 2:**
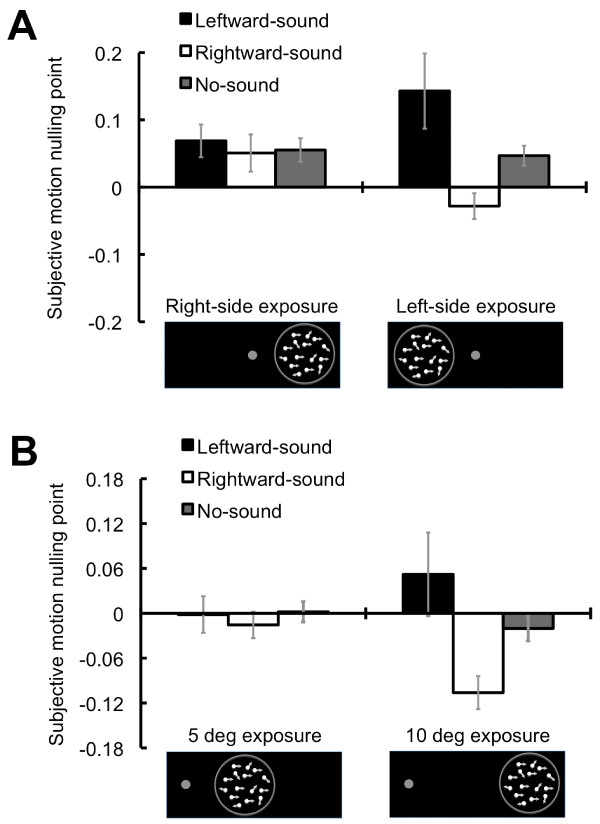
**Results for selectivity of the visual field**. (A) SMNPs for the left visual field. The ANOVA with exposures (2; right-/left-side) × auditory conditions (3) showed the significant interaction effect between the factors (*F*(2, 16) = 5.68, *p *< .05). As for a simple main effect of the auditory conditions in the left-side exposure (*F*(2, 32) = 12.60, *p *< .001), the post hoc test revealed that the SMNPs were different among the auditory conditions (*p *< .05). In contrast, the simple main effect of the auditory conditions in the right-side exposure was not significant (*F*(2, 32) = .16, *p *= .85). (B) SMNPs at 10 deg of eccentricity. The ANOVA with exposures (2; 5/10 deg) × auditory conditions (3) showed the significant interaction effect between the factors (*F*(2, 16) = 8.39, *p *< .005). With regard to a simple main effect of the auditory conditions at 10 deg of eccentricity (*F*(2, 32) = 21.12, *p *< .001), the post hoc test revealed that the SMNPs were different among the auditory conditions (*p *< .05). The simple main effect of the auditory conditions at 5 deg of eccentricity was not significant (*F*(2, 32) = .29, *p *= .75). Error bars denote the standard error of the mean.

The auditory stimuli might simply bias the participant's judgment; to test this possibility, we examined whether the auditory effect was observed at the retinal position where the visual and auditory stimuli were not exposed. The test session was conducted at the left visual field (5 deg of eccentricity) after the exposure of the right visual field. No auditory effect was observed, suggesting that the effect of the exposure is well observed only at the visual field where the exposure was provided. Indeed, the effect was seen after the exposure of the left visual field (Figure 2A). We further confirmed that the selectivity could be observed within a hemifield: The tests were conducted at 10 deg of eccentricity in the right hemifield. The sounds did not have the effect after the exposure at 5 deg of eccentricity. In contrast, a clear auditory effect occurred after the exposure at 10 deg of eccentricity (Figure 2B). These results indicate that the current results cannot be explained only by the sound-induced bias on the participant's judgment.

## Discussion

The present study focused on the sound-contingent visual motion aftereffect; arbitrary sound can induce visual motion perception in the previously presented manner after the short-term exposure of paired sound and visual motion information. The arbitrary sound frequency and visual motion direction may associate rather easily after the prolonged exposure of these stimuli. We also found that the sound-contingent visual motion aftereffect persists for at least 2 days. These results indicate that the short-term presentation of paired sound and visual motion information was enough to establish a long-term contingent motion aftereffect. Further, the sound-contingent motion aftereffect was not transferred between the visual fields. This implies that the sound and visual motion information is associated at relatively early stages of perceptual processing.

**Figure 3:**
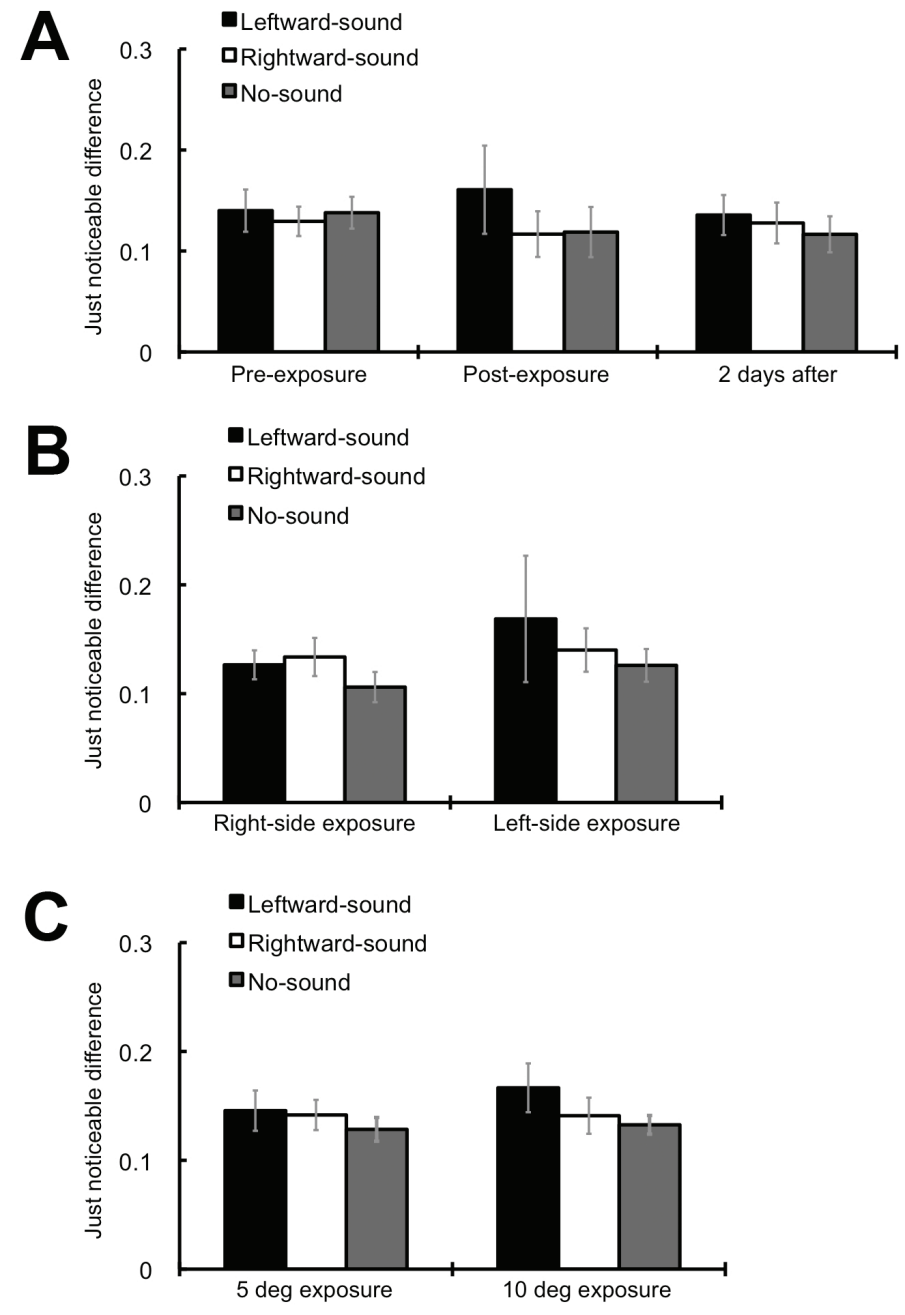
**Just noticeable differences**. (A) Just noticeable differences (JNDs) for exposure effects. The ANOVA revealed no significant main (tests: *F*(1,8) = .04, *p *= .85; auditory conditions: *F*(2,16) = 2.52, *p *= .11) and interaction effects (*F*(2,16) = 1.28, *p *= .30) with regard to the comparison between the pre and post tests. A similar result was obtained for the test session 2 days after the exposure; the main (tests: *F*(1,8) = .07, *p *= .42; auditory conditions: *F*(2,16) = .93, *p *= .41) and interaction effects (*F*(2,16) = .83, *p *= .45) were not significant. (B) JNDs for the left visual field. The ANOVA revealed no significant main (exposures: *F*(1,8) = 1.71, *p *= .22; auditory conditions: *F*(2,16) = .99, *p *= .39) and interaction effects (*F*(2,16) = .35, *p *= .71). (C) JNDs at 10 deg of eccentricity. The ANOVA revealed no significant main (exposures: *F*(1,8) = 1.01, *p *= .35; auditory conditions: *F*(2,16) = 1.91, *p *= .18) and interaction effects (*F*(2,16) = .81, *p *= .46). Error bars denote the standard error of the mean.

One might assume that the tones simply biased the participants' responses or judgments. A previous study reported that auditory motion information affected the judgments of perceived visual motion direction in a global visual motion display only when the coherence of the visual local motion signal was considerably low [6]. This tendency could indicate the presence of the response or decisional bias, since auditory information was utilized for making decisions only when the visual motion direction was difficult to discriminate on its own [7]. Thus, given that the response/decisional bias existed, the slope of the psychometric functions should become less steep, especially when the global motion display contained lower coherences. We confirmed that this was not the case by calculating the slope of psychometric functions (just noticeable differences: JNDs) using the following formula: (75% threshold - 25% threshold)/2. The ANOVA did not find significant effects in any of the data (see Figure 3). In addition, as clearly illustrated in Figure 1B, the tones paired with the rightward/leftward visual motion perceptually cancelled out the opposite visual motion signal and induced the consistent motion perception of the paired global visual motion even when there were relatively sufficient visual motion signals. We would like to note also that the effects of the tones were firmly limited to the visual field where the exposure was provided, indicating the involvement of relatively low perceptual processing. These results indicate that the effects of the exposure cannot be simply explained by the response/decisional bias.

Previous studies have reported on the audiovisual interaction in motion perception. While the effects of visual information on auditory motion perception have been primarily reported [8-10], recent studies have shown both the modulatory [11-13] and driving/inducing [14,15] effects of auditory information on visual motion perception, even for visual global motion displays [6,7,16]. It is notable that a transient sound modulated ambiguous visual motion perception to disambiguate one by capturing the temporal positional information of a moving visual stimulus [13] and that sounds containing motion information triggered [14-16] or altered [6,7,16] visual motion perception. The audiovisual interactions in motion aftereffect were also reported. For instance, adaptation to visual stimuli moving in depth induced auditory motion aftereffect in terms of changes in perceived sound intensity [17]. It was also reported that visual motion information modulated auditory motion aftereffect [18]. Adaptation to auditory motion also induced the visual motion aftereffect, although the effect was limited to the vertical plane [19]. It is worth noting about these findings that the auditory or visual adapter was in motion. Contrary to the above-mentioned findings, the sounds used in this study had no spatiotemporal or motion information: The tones containing only arbitrary frequency information could induce visual motion perception after the short-term exposure of paired tones and visual motion. On the basis of these facts, we regard our findings as showing the audiovisual contingent aftereffect.

A previous study showed that tones could induce visual apparent motion perception to a static blinking visual stimulus after a prolonged exposure to alternating left-right visual stimuli together with high or low frequency tone, wherein the onset of each tone was synchronized with that of the visual stimuli [20]. However, it remains unclear whether the tones were associated with visual apparent motion or with positional information (left or right) of the visual stimuli. In our study, motion perception was derived from the integrated visual motion signal of dots in global motion display, and not from the positional information of each dot. Therefore, our results clearly demonstrated audiovisual contingent motion aftereffect: Single tone can be directly associated with motion directional information (leftward or rightward) and can act as a driver for visual motion perception.

It should be noted that some phenomenal aspects of our findings differ from the unimodal contingent motion aftereffects; for instance, in our study, the sound-contingent visual motion aftereffect was positive (i.e., a tone associated with leftward motion induces leftward motion perception), whereas it was negative in the unimodal contingent aftereffects (i.e., a stimulus associated with leftward motion induces rightward motion perception). Studies on audiovisual association learning also reported a positive effect. After the presentation of paired auditory and visual moving stimuli, auditory motion information was found to improve the discrimination performance for visual motion [21]. The audiovisual association learning in motion perception was observed only when spatiotemporal [22] or situational consistency [23] was maintained between the stimuli. However, in the present study the arbitrary sound contained no explicit spatiotemporal or motion information. Moreover, a few minutes' observation of the stimuli without any task could induce a motion aftereffect in our study, while the association learning usually needs explicit training wherein the participants are engaged in required tasks [21-23]. These points suggest that the findings of the present study cannot be fully explained by association learning.

In contingent motion aftereffect, new cortical units or representations are established by perceptual learning [2,3]. In line with the previous study showing positive audiovisual temporal aftereffects [24], the findings of our study indicate that perceptual systems can rapidly form associations between single sound and visual motion information and establish a new neural representation between auditory and visual modalities with respect to motion perception. The negative aftereffect seen in the unimodal contingent aftereffects suggests that a prolonged exposure of paired stimuli (red contraction and green expansion) establishes cross-association in new neural representations (red expansion and green contraction). In contrast, the positive aftereffect seen in the sound-contingent motion aftereffect indicates that the exposed audio-visual information is straightforwardly bound together and establishes new neural representations. It is noteworthy that the unimodal contingent motion aftereffects require more than 10 minutes of exposure, while a few minutes of exposure can associate sound with visual motion in the sound-contingent motion aftereffect. Future research should focus on the differences in the functional characteristics and underlying mechanisms between audiovisual and unimodal contingent aftereffects.

## Conclusion

The current study focused on the sound-contingent motion aftereffect; the presentation of paired arbitrary sounds and motion directional information for few minutes resulted in auditory-induced effect on visual motion directional perception. This auditory effect was positive as it replicated the previous paired presentation, and it lasted for at least 2 days. The findings of our study indicate that the perceptual systems can rapidly form a direct association between a sound without explicit spatiotemporal or motion information and visual motion information and that they can establish a new neural representation between auditory and visual modalities.

## Authors' contributions

SH, WT, and YS conceived of the study and participated in its design. SH, WT, MK, and YS performed the experiments and participated in data analysis and interpretation. SH and YK drafted the manuscript. WT and MK contributed to discussion and revision of the manuscript. All authors read and approved the final manuscript.
